# Bias in Observational Studies of the Association between Menopausal Hormone Therapy and Breast Cancer

**DOI:** 10.1371/journal.pone.0124076

**Published:** 2015-05-04

**Authors:** Per-Henrik Zahl, Jan Mæhlen

**Affiliations:** 1 Norwegian Institute of Public Health, Oslo, Norway; 2 Ullevål University Hospital, Oslo, Norway; Baylor College of Medicine, UNITED STATES

## Abstract

**Background:**

During the period 1985-2000 the breast cancer incidence rates increased 50% in the age group invited to mammography screening in Norway and Sweden. Simultaneously, use of hormone replacement treatment therapy (HT) increased 5 times. Several influential observational studies showed that HT was associated with 50% to 100% increased risk of breast cancer and most for those using combined (estrogen plus progestin) hormone replacement therapy (CHT). In contrast, the randomized WHI trial reported that CHT increased the risk by 10% for those not having previously used hormones and 24% when including previous users in the analyses. In another randomized trial, estrogen use only was not associated with any increased risk at all. After the WHI trial was published in 2003, use of HT dropped 70% within 5 years in Norway and Sweden while breast cancer rates were essentially unchanged. After 2008, HT use has dropped further and breast cancer incidence rates have started increasing again. The study objective is to calculate and to explain potential bias in the observational study design.

**Methods and Findings:**

Here we use data from the randomized WHI trial and analyze these data as done in the observational studies to calculate the magnitude of the potential biases in the observational study design. Time varying effect of hormones and categorization of the follow-up time may increase the hazard ratio for long-term users from 1.10 to 1.48. Selective retrospective reporting of hormone use may further increase the hazard ratio to 1.68.

**Conclusions:**

We suggest that the mechanism causing higher hazard ratio of breast cancer (compared to the observational studies) is the time-varying effect of CHT on the breast cancer risk and selective retrospective reporting of hormone use. Other risk factors for the increase in breast cancer risk in the age group 50-69 years should be considered, for example, overdiagnosis.

## Introduction

In the period 1985–2000 breast cancer incidence increased about 50% in the age groups invited to screening [1]. Simultaneously several important observational studies reported large increases in breast cancer risk among women using hormone replacement therapy (HT); the Million Woman (MW) study [2] reported a 100% increase of invasive breast cancers among current users of combined (estrogen plus progestin) hormone replacement therapy (CHT) and similar increases were observed in two observational studies from Norway with estimated population attributable fractions of 14% and 27%, respectively [2–4]. Use of estrogen only was associated with a lower risk. After screening was introduced in Norway, HT use dropped 70% within 5 years, while breast cancer incidence rates were constant [5–7]. A similar trend was observed in Sweden [7,8]. If CHT was the main cause for the incidence increase before year 2000, then we would expect breast cancer rates to fall dramatically when 70% of women suddenly stop using CHT. Moreover, in the period 2007–12 breast cancer risks in the screened age group has increased further (from 275 per 100,000 to about 320 per 100,000 women in Norway and Sweden) [7,8], while use of CHT has kept on falling [5].

In contrast to the observational studies, the Women’s Health Initiative (WHI) trial reported only 24% increased risk of invasive breast cancer after on average 5.6 years follow-up among women randomized to CHT [9]. Among women with no exposure to hormone therapy before randomization, there was only 10% incidence increase in the treatment group. The WHI trial reported no significant effect of estrogen only on the breast cancer incidence [10].

Another important observation in the WHI trial was that CHT had a biphasic effect on the breast cancer incidence. Compared to the control group, the incidence was temporarily reduced by 41% in the first 2 years of the follow-up (calculated from table 2 in [9]) for women with no prior exposure to CHT. It was also a significant time-varying effect for those with a history of prior exposure to CHT. The most likely explanation of this time-varying effect is that CHT increases the breast tissue density causing reduced sensitivity at mammography leading to an initial reduction in the detection of small breast tumors [11]. After 2 years follow-up, a strong increase in the numbers of breast cancer was observed in the CHT group and the tumors were also on average larger. A substantial part of the increase after 2 years follow-up may be accumulated tumors that have escaped detection during the first 2 years.

Another problem in observational studies is that some people reporting no CHT use at study start will start using hormones during the follow-up. Usually, unreported CHT use among women assumed not to be using CHT (typically the reference group in an observational study), leads to an underestimation of the drug effect. However, in this case and very contra-intuitively, it causes overestimation because an influx of women who use CHT for less than three years and who have lower breast cancer risks than no-users.

Retrospectively reporting the length of drug use may be associated with information bias [12]. Finally we show that information bias also may lead to overestimation of the CHT effect in an observational study and this overestimation is also related to the biphasic effect of CHT.

The objective is to quantify the sizes of bias in observational studies of CHT and breast cancer using 3 different realistic scenarios all with 3 years follow-up. We use data from the more reliable randomized WHI trial [9] to calculate the magnitude of the potential biases in the MW study and other similar studies [2–4].

## Data and Methods

**Fig 1:**
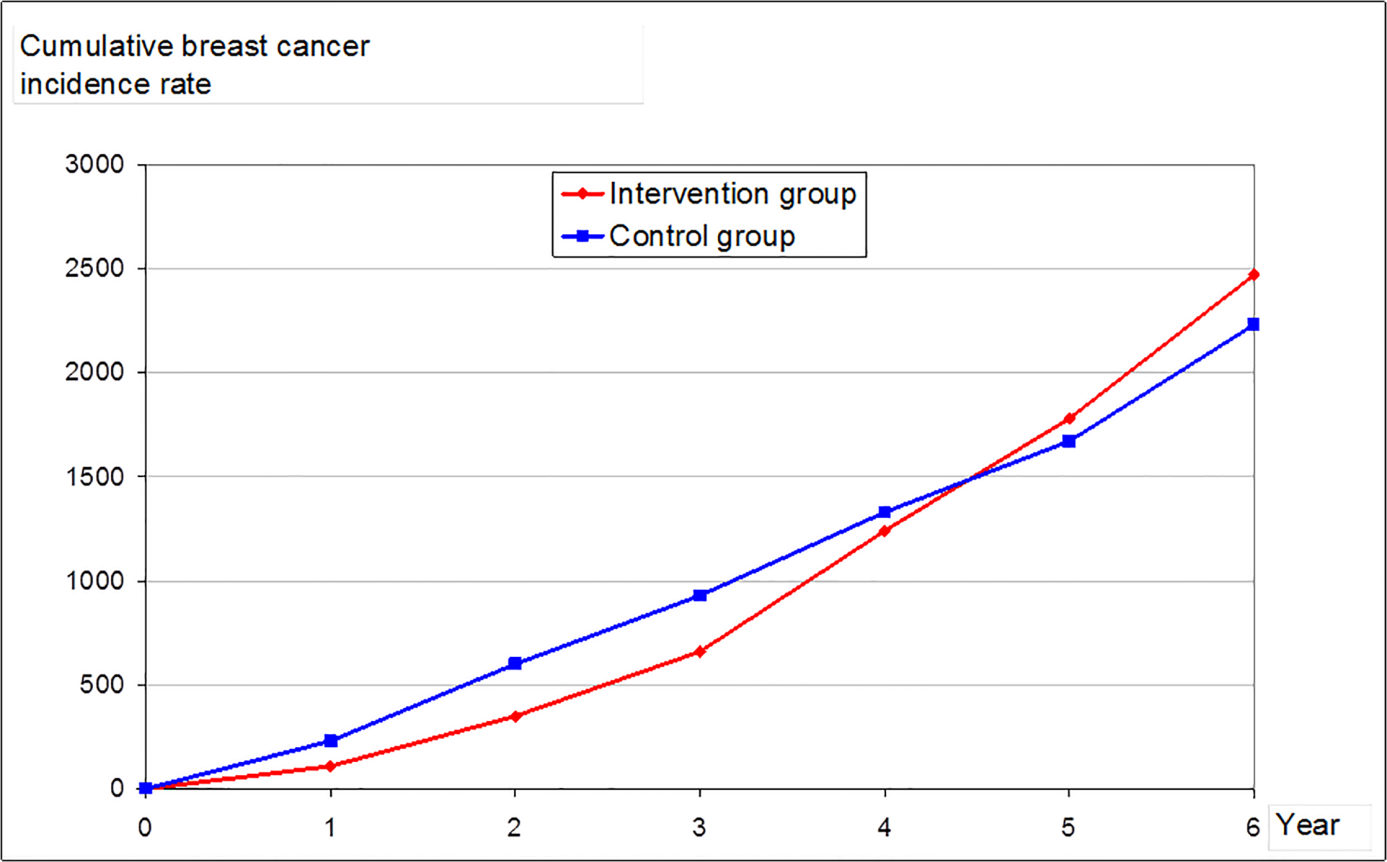
Cumulative breast cancer incidence rates for women in the WHI trial that had not used CHT previous to the randomization.

In the WHI trial 8506 women were randomized to CHT and 8102 to placebo treatment [9]. After excluding 4304 women with exposure to CHT before randomization, there were 6277 in the CHT group and 6020 in the placebo group. In the latter subgroups, there were diagnosed 41 breast cancers in the CHT group and 55 in the placebo group in the first 3 years of follow-up. The corresponding cumulative breast cancer incidence rates were 660 and 930 per 100,000. In the next 3 years of the follow-up, there were another 100 breast cancers in the CHT group and 66 in the placebo group and the corresponding cumulative rates after 6 years were 2470 and 2230 respectively (Fig 1). The hazard ratio (HR) among CHT users were 0.71 (660/930) and 1.10 (2470/2230) after 3 and 6 years follow-up, respectively [9]. Below we assume these time-varying incidence rates are true and unbiased.

**Fig 2:**
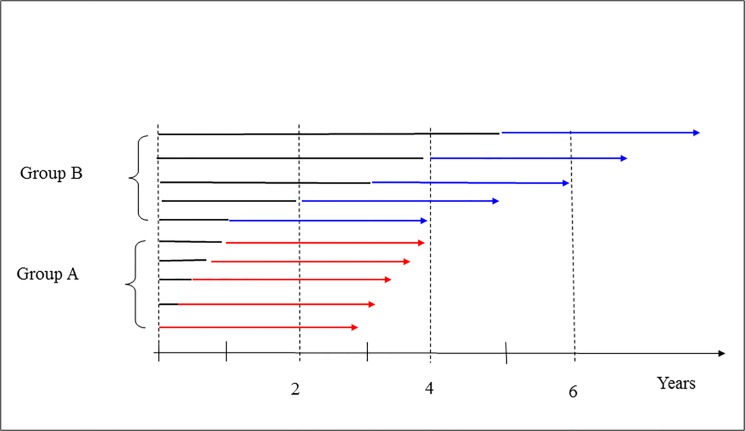
Women who have used CHT in 3 to 4 years are referred to as Group A; women who have used CHT in 4 to 9 years are referred to as group B. The arrows indicate the individual follow-up periods from first starting using CHT. In a randomized trial women are followed from time zero to the end of the arrows. In the observational studies women are only followed in the red and blue coloured part of the arrows. If the breast incidence is time-varying, then this will lead to systematic shift of diagnosis from the black part of the arrows to the coloured part of the arrows because of delay in time of diagnosis. This is leading to an over-estimation of the hazard rates in the period indicated by red and blue colours.

Based on the incidence rates in the WHI trial, we constructed 3 data sets (or scenarios) which we later used in observational studies with similar follow-up and categorization of CHT users as reported in the observational studies [2–4]. First note that the average follow-up time is 3 years the observational studies but 5.6 years in the randomized trial [2–4,9]. At study start we assume that women could have used CHT for 0–1 or 1–5 years in our fictional observational study. The average time on CHT for women reporting 0–1 and 1–5 years use at study start and with 3 years follow-up is therefore 3.5 and 6 years respectively in our data set (Fig 2).

When constructing the data set for our observational study, we assumed that the duration of CHT use at study start was uniformly distributed over 0–12 months for the short-time users (group A in Fig 2) and over 1–5 years for long-time users (group B in Fig 2). Thus, the difference in the mean age for groups A and B is 2.5 years (which is the same as the difference in mean duration time of CHT use in the two groups). The maximum follow-up in the WHI trial was 7 years and with a joint incidence estimate for the 6-7th year. The constructed population data included women who had used CHT for 0–5 years at entry and with 3 years follow-up and the maximum time on CHT is 8 years. Therefore, we had to assume that the incidence rate in the 8th year of follow-up was as in the 6-7th year. We had no data to study the bias for women who had used CHT for longer than 5 years at study start.

The numbers of breast cancers in the constructed data sets are given in Tables 1–3, and the numbers are calculated from the Table 2 in Chlebowski et al [9]. In the second column in Table 1 (scenario 1), the numbers of breast cancers for short time CHT users (group A) are given: the numbers in the first year of the follow-up in Table 1 is the average number of breast cancers in the 2 first years of the follow-up in the WHI trial (this is calculated as (7+15)/2 = 11); the second year of follow-up in Table 1 is the average number of breast cancers in the second and third year of the follow-up in the WHI trial (this is calculated as (15+19)/2 = 17); and the third year of follow-up is the average of the third and fourth year of follow-up in the WHI trial (this is calculated as (19+35)/2 = 27). In the fifth column in Table 1 the numbers of breast cancers for long time CHT users (group B) are given; the number of breast cancers in the first year of follow-up in Table 1 is calculated as the average number of breast cancers in the period 2.5–6.5 years in the WHI trial and this is calculated as (0.5∙15+19+35+28+0.5∙37)/4 = 27; the number of breast cancers in the second year of the follow-up is calculated as the average number of breast cancers in the period 3.5–7.5 years in the WHI trial and this is calculated as (0.5∙19+35+28+37+0.5∙37)/4 = 32; and the number of breast cancers in the third year of the follow-up in Table 1 is calculated as the average number of breast cancers in the period 4.5–8.5 years in the WHI trial and this is calculated as (0.5∙35+28+37+37+0.5∙37)/4 = 34.5. The numbers in the third and sixth columns in Table 1 are calculated analogously as above but using data from the placebo group. Note that the time-varying incidence rates automatically adjust for the time variable in the first and forth columns.

**Table 1:** (Scenario 1) The number of breast cancer cases in the intervention group for the short term users (Group A) and long term users (Group B).

Time	Breast cancers		Time	Breast cancers	
	Group A	Control		Group B	Control
1	11	18	3.5	27	20.75
2	17	20.5	4.5	32	22.13
3	27	21	5.5	34.5	23.38

The numbers of CHT users were 6277, and there were 6020 in the control group. The time variable is time since randomization.

**Table 2:** (Scenario 2) The number of breast cancer cases in the intervention group for the short term users (Group A) and long term users (Group B).

Time	Breast cancers		Time	Breast cancers	
	Group A	Control		Group B	Control
1	11	15.3	3.5	27	17.64
2	17	17.43	4.5	32	18.81
3	27	17.85	5.5	34.5	19.87

The numbers of CHT users were 6277, and there were 6020 in the control group. The time variable is time since randomization.

**Table 3:** (Scenario 3) The number of breast cancer cases in the intervention group for the short term users (Group A) and long term users (Group B).

Time	Breast cancers		Time	Breast cancers	
	Group A	Control		Group B	Control
1	19	25.5	3.5	37.88	25.13
2	27.5	27	4.5	42.25	25.63
3	36.5	24.5	5.5	44.38	26.75

The numbers of CHT users were 8506, and there were 8102 in the control group. The time variable is time since randomization.

Sales figures of CHT in Norway increased from 10 to 40 Defined Daily Doses (DDD)/day per 1000 women in the period 1991–2000 [5,7]; i.e. the user prevalence increased from 5% to about 20% if all hormones were used in the age group 50–69 years. If we also assume women in the age group 50 to 69 years use CHT for 2 years on average, then there must be about 9% new users annually to increase the user frequency from 5% to 20% over 5 years. Thus, it is reasonable to assume there are 10% new users every year in the non-user group; i.e. there are 30% new users over 3 years and we assume these women have 50% reduced risk of breast cancers because they are short term users. In Table 2 (scenario 2) we have adjusted the number of breast cancers in the control group for new users after study start by multiplying the numbers in columns 3 and 6 in Table 1 by 0.85.

In Table 3 (scenario 3), we also assumed that a similar number of women as in the WHI trial had used CHT before entry into the observational study. In second column the numbers of breast cancers for short time CHT users (group A) are given: the numbers in the first year of the follow-up in Table 3 is the average number of breast cancers in the 2 first years of the follow-up in the WHI trial (this is calculated as (12+26)/2 = 19); the second year of follow-up is the average number of breast cancers in the second and third year of the follow-up in the WHI trial (this is calculated as (26+29)/2 = 27.5); and the third year of follow-up is the average of the third and fourth year of follow-up in the WHI trial (this is calculated as (29+44)/2 = 36.5). In the fifth column in Table 3 the numbers of breast cancers for long time CHT users (group B) are given; the number of breast cancers in the first year of follow-up in is calculated as the average number of breast cancers in the period 2.5–6.5 years in the WHI trial and this is calculated as (0.5∙26+29+44+43+0.5∙45)/4 = 37.88; the number of breast cancers in the second year of the follow-up is calculated as the average number of breast cancers in the period 3.5–7.5 years in the WHI trial and this is calculated as (0.5∙29+44+43+45+0.5∙56)/4 = 42.25; and the number of breast cancers in the third year of the follow-up in Table 3 is calculated as the average number of breast cancers in the period 4.5–8.5 years in the WHI trial and this is calculated as (0.5∙44+43+45+45+0.5∙45)/4 = 44.38. The numbers in the third and sixth columns in Table 3 are calculated analogously as above but using data from the placebo group.

We used 6277 as the number of individuals at risk in groups A and B, and 6020 for the control groups in Tables 1 and 2. In Table 3, we used 8506 (CHT users) and 8102 (non-users) as the number of individuals, respectively.

We used Poisson regression to calculate HR in the observational study with 3 year follow of women who had no prior CHT-exposure (Table 4) first using data in Table 1 (scenario 1). We then repeated the regression analysis under the assumption that during each year of the follow-up, 10% of the non-users started using CHT (using data in Table 2, scenario 2). Finally we did the analysis under the assumption that the incidence rates in the constructed data set were identical to that observed for all 16 608 women in the WHI trial (scenario 3); i.e. we assumed that 26% of the women had previously used CHT but did not correct for previous use (using data in Table 3). We also calculated the numbers of breast cancers when assuming that only 5% of the non-users started using CHT after the start of the follow-up; the calculated HRs for these numbers are given in parenthesis in Table 4.

**Table 4:** Hazard ratios of breast cancer when data from the randomized WHI study are analyzed as a fictive observational study.

Duration of CHT use at baseline	Model		
	1	2	3
0-1 year	0.96	1.14 (1.04)	1.30 (1.21)
1-5 years	1.27	1.48 (1.37)	1.68 (1.69)

Model 1 is a regression model with 3 year follow-up (scenario 1); model 2 is the same model assuming that 10% starts using hormones every year in the non-user group (scenario 2); in model 3 (scenario 3) it is assumed that the incidence rates in the fictive study were identical to that observed for all 16 608 women of the WHI trial. In parenthesis we also give HR when assuming that 5% start using hormones every year in the non-user groups.

In the observational studies, many women in the non-user group started using CHT during the follow-up period [2–4]. In the WHI trial the control group received placebo treatment, and it was therefore assumed that very few started using CHT in the follow-up period. By using the rates in the WHI trial, we have indirectly assumed that the relative number of women who stopped using CHT is as in the WHI trial [9].

## Results

In the constructed observational study, the HRs of breast cancer for women who had used CHT for less than 1 year and for 1–5 years at entry were 0.96 and 1.27, respectively (Table 4, model 1), while the unbiased estimates were 0.70 and 1.10. The average duration of CHT for the long-term users is 6 years (compared to 5.6 years in the WHI study). Assuming that 10% of the non-users start using CHT every year during the 3-year follow-up period, the HRs were 1.14 and 1.48, respectively (Table 4, model 2). Under the assumption that the incidence rates in our constructed observational study were identical to that observed for all women in the WHI trial, the calculated HRs were 1.30 and 1.68, respectively (Table 4, model 3).

We also calculated HR when only 5% of the non-users started using CHT in each year of the follow-up period (Table 4). The HRs for those who had not used CHT prior to randomization were 1.04 (short term users) and 1.37 (long-term users). The corresponding HR when also including those who had used CHT prior to randomization were 1.21 and 1.69 respectively.

A simple calculation demonstrates that the numbers in Table 1 can be analysed as in the randomized WHI trial and with a similar result: For group A (and the corresponding control group), each number is the average over 2 years; this means that the sum after 3 years is the number of breast cancers from 0.5 to 3.5 years in the WHI trial. The sum over 3 years for group A is 55 and for group B it is 93.5. For the respective control groups the sums are 59.5 and 66.26, respectively. This gives HR = ((55+93.5)/6277)/((59.5+66.26)/6020) = 1.13 after 6 year follow-up, which is very close to the 1.10 in the WHI trial [9].

## Discussion

We show that the discrepancy between the randomized WHI trial and the observational studies can be explained by bias in observational studies with short follow-up. First, when analyzing the data in the WHI trial using an observational study design, HR increases from 1.10 to 1.27 in the long-term users (model 1). The difference in HRs is caused by differences in the start of follow-up and in the length of the follow-up: all women have used CHT for 6 years in the randomized study design and follow-up starts from time of randomization, while in the observational design (model 1) long-term users have used CHT on average for 6 years (varying from 4 to 8 years) but the follow-up starts 1–5 years after randomization and the follow-up is 3 years. A combination of time-varying incidence rates and the influx of new CHT users in the non-user group after study start decreases the incidence in non-users leading to a further increase of the HR from 1.27 to 1.48 (model 2). Usually, drug use in the control group leads to an underestimation of the difference; however, in this case the effect is the opposite of what is intuitively expected: the difference is overestimated. Furthermore, retrospectively recording the length (leading to information bias) of the CHT use causes a further increase from 1.48 to 1.68 (model 3). The WHI study also reported higher HR when adjusting for low adherence, which may explain further differences; however, we are not able to quantify these.

An alternative interpretation of model 3 is that the sum of a time-varying affect of CHT on the incidence rate (no effect in the first 2 years and then a strong effect in the next 4 years with a 24% incidence increase after 6 years follow-up) and with an influx of new CHT users in the non-user group after study start causes the HR to increase from 1.24 to 1.68.

Our assumption that long term users have higher risk than short term users is crucial for our analysis and it is actually supported by the observational studies too [2–4]. However, after about 5 years use there is little evidence for any further increase in risk [2]. Therefore, the bias will decline when the follow-up increases to more than 3 years. Our analysis is not very sensitive to the proportion of new users: In parenthesis in Table 4 we also give HR when assuming that only 5% of non-users start using CHT every year. Our estimates are almost not affected by changing the annual numbers of new users from 10% to 5%, showing that with 3 years follow-up it is the biphasic rate rather than the prevalence of new users that is causing bias.

From year 2002 to 2007 use of HT (including CHT) dropped 70% to the level of 1990 in Norway, while the breast cancer incidence rate was essentially unchanged [5,7]. In the same period the decline in the breast cancer incidence in the UK was about 5% in the age group 50 to 59 years [13]. The decline in breast cancer incidence in Sweden was 7.5% for the age group 50–69 years in the period 2002–7 [7,8]. After 2007 breast cancer incidence rates in the screened age groups in Norway and Sweden have increased another 15% [6,8] and HT use have kept on falling [5] which additionally underlines there must other causes for the increase of breast cancer in the last 3 decades, for example overdiagnosis [1].

One may argue that there are major differences in the age composition in the studies. Women in the WHI trial were older than women in the observational studies; however, there were no significant age difference in the WHI trial [9]. It has been suggested that the difference may be explained by “gap time” effect [14]; the gap time is time from menopause to first use of CHT. The gap time model is sensitive to modeling assumptions and furthermore, because two thirds of data comes from the observational WHI study, the model is essentially an observational study.

In our regression analyses we have adjusted for a 2.5 years difference in age between the control group and the CHT users groups. Without age adjustment the HR would have been about 0.1 higher for women that had used CHT for 1 to 5 years at baseline, but the HR would have been lower for women who had used CHT for 0 to 1 years.

If for example 20% of women aged 50 to 69 years were using CHT and the breast cancer incidence increases is as in the WHI trial [9], the total incidence increase will be about 5% if all women use it for 6 years or more. When women stop using CHT, the incidence rate dropped back to normal within 1–2 years in the WHI trial [15] and the same was observed in the MW study [2].

The average length on HT varies between studies. In the WHI observational study women had used CHT for on average 6.9 years at entry and were followed-up in 7 years [14]. This is much longer than in the randomized WHI trial [9] and the MW study [2]. Furthermore, there were 145 000 past users and 278 000 current users (median time on HT is 5 years) in the MW study. In the UK, the HT use is dominated by women who used HT for less than 5 years [2]. A much smaller incidence decline in Europe than in US may be explained by differences in how long women have used HT.

In contrast to Europe, a much larger decline was reported in the US after the release of the WHI study [16]. But it is also possible that less frequent use of mammography may have contributed to the decline. Breen et al. [17] reported a decline from 78% to 72% in the US mammography activity after age 50 occurring about year 2002. Glass et al. [18] also reported that a decline in mammography activity may explain some of the drop. In another US study, Kerlikowske et al. [19] suggested that a decline in screening mammography is unlikely to account for the recent decline in US breast cancer incidence rates. However, Kerlikowske et al. [19] did not report mammography screening rates, and changes in these rates may explain the change in the breast cancer rate because of overdiagnosis related to screening.

Ioannidis [20,21] noted that contradictions and initially stronger effects are frequent in highly cited clinical research/interventions and their outcomes, especially in non-randomized studies, and suggested to study possible causal mechanisms. Our study shows that a realistic combination of time-varying rates observed in a randomized trial may yield similar results as those found in recent observational studies. We show that time-varying hormone effects triple the effect of CHT in an observational study design and this may explain Ioannidis observations. Consequently the findings of the observational studies of increased incidence rates of breast cancer due to use of CHTs may be spurious and biased.

### Ethics and informed consent

No approval from an ethical committee or informed consent was needed.
